# Is Asthma Related to Choroidal Neovascularization?

**DOI:** 10.1371/journal.pone.0035415

**Published:** 2012-05-02

**Authors:** Yaoyao Sun, Wenzhen Yu, Lvzhen Huang, Jing Hou, Peihua Gong, Yi Zheng, Mingwei Zhao, Peng Zhou, Xiaoxin Li

**Affiliations:** 1 Department of Ophthalmology, Peking University People’s Hospital, Beijing, China; 2 Key Laboratory of Vision Loss and Restoration, Ministry of Education, Beijing, China; 3 Department of Respiration, Peking University People’s Hospital, Beijing, China; 4 Department of Immunology, School of Basic Medical Sciences, Peking University, Beijing, China; 5 Department of Ophthalmology, Eye and ENT, Hospital Affiliated to Fudan University, Shanghai, China; Institut de la Vision, France

## Abstract

**Background:**

Age-related degeneration(AMD) and asthma are both diseases that are related to the activation of the complement system. The association between AMD and asthma has been debated in previous studies. The authors investigated the relationship between AMD and asthma systemically.

**Principal Findings:**

The epidemiological study showed that asthma was related to choroidal neovascularization(CNV) subtype(OR = 1.721, P = 0.023). However, the meta-analysis showed there was no association between AMD and asthma. In an animal model, we found more fluoresce in leakage of CNV lesions by FA analysis and more angiogenesis by histological analysis in rats with asthma. Western blot demonstrated an elevated level of C3α-chain, C3α’-chain and VEGF. After compstatin was intravitreally injected, CNV leakage decreased according to FA analysis, with the level of C3 and VEGF protein decreasing at the same time.

**Significance:**

This study first investigated the relationship between AMD and asthma systematically, and it was found that asthma could be a risk factor for the development of AMD. The study may provide a better understanding of the disease, which may advance the potential for screening asthma patients in clinical practice.

## Introduction

Age-related macular degeneration (AMD), the most common cause of irreversible blindness in the elderly population in many countries, affects the macular area of the retina [1], [2]. There are two major clinical phenotypes of AMD–a nonexudative type(dry AMD) and an exudative type (wet AMD) [3]. During the development of AMD, choroidal neovascularization (CNV), or the development of new pathological blood vessels, is the major cause of vision loss [4]. The pathogenesis of CNV is poorly understood. As a complex disease, multiple environmental and genetic risk factors for CNV have been identified [5]–[9]. Studies recently indicated that inflammation, especially the alternative complement pathway, plays a fundamental role in the development of CNV [10]. Additionally, genetic evidence has identified variations in multiple genes involved in the complement cascade, including complement factor 3 (C3), complement factor H (CFH), complement factor Band complement factor 2 (C2), associated with AMD [11]–[13]. Apart from the complement system, other angiogenic stimuli have been reported to take part in the development of CNV, with the most important one being vascular endothelial growth factor [14].

Bronchial asthma (BA) is an inflammatory disorder of the airways characterized by airway hyper responsiveness and reversible airway obstruction [15]. Like CNV, BA is a multifunctional disorder with both environmental and genetic factors contributing to its development. Recent studies reveal that the complement system plays a crucial role in the development of immunological responses in BA by initiating and/or amplifying airway inflammation [16], [17]. Variations of genes in the complement system have been shown to confer susceptibility to BA, including complement factor 3(C3) and complement factor 4(C4).

Because the mechanisms of development for both CNV and asthma are alike, we hypothesize that there is a relationship between these two diseases. During the past decade, several population-based studies reported that a history of asthma is associated with a high risk of developing CNV. However, other studies have also shown no association between asthma and CNV. Therefore, the association between these two diseases remains unknown.

In this study, we examined the cross-sectional relationship between asthma and CNV in a population-based sample of the mongoloid race in China. We then carried out a meta-analysis on all currently available studies to estimate the strength of a history of asthma being associated with CNV. Furthermore, a rat model of CNV induced by laser was developed in rats with asthma to investigate whether asthma is a risk factor for CNV and the potential mechanism of association.

## Results

### An Epidemiological Study Demonstrating the Association between CNV and Asthma

An epidemiological study was performed to determine if there is a correlation between a history of asthma and CNV. Of the 462 AMD patients, asthma was present in 47(10.17%) patients, whereas out of the 502 healthy controls, 31(6.18%) had asthma. The association of asthma and CNV was statistically significant (OR = 1.721, P = 0.023). As a result, asthma was found to be related to CNV (Tab.1).

**Table 1:** The epidemiological study among Chinese people.

	Asthma (%)	Non-asthma(%)	χ^2^	P	OR	95%CI
CNV	47 (10.17)	415 (89.83)	5.171	0.023*	1.721	(1.073,2.759)
Non-CNV	31 (6.18)	471 (93.82)				

Patients with a history of asthma that developed CNV compared with controls in our epidemiological study.

### Meta-analysis Showing no Association between CNV and Asthma

**Figure 1:**
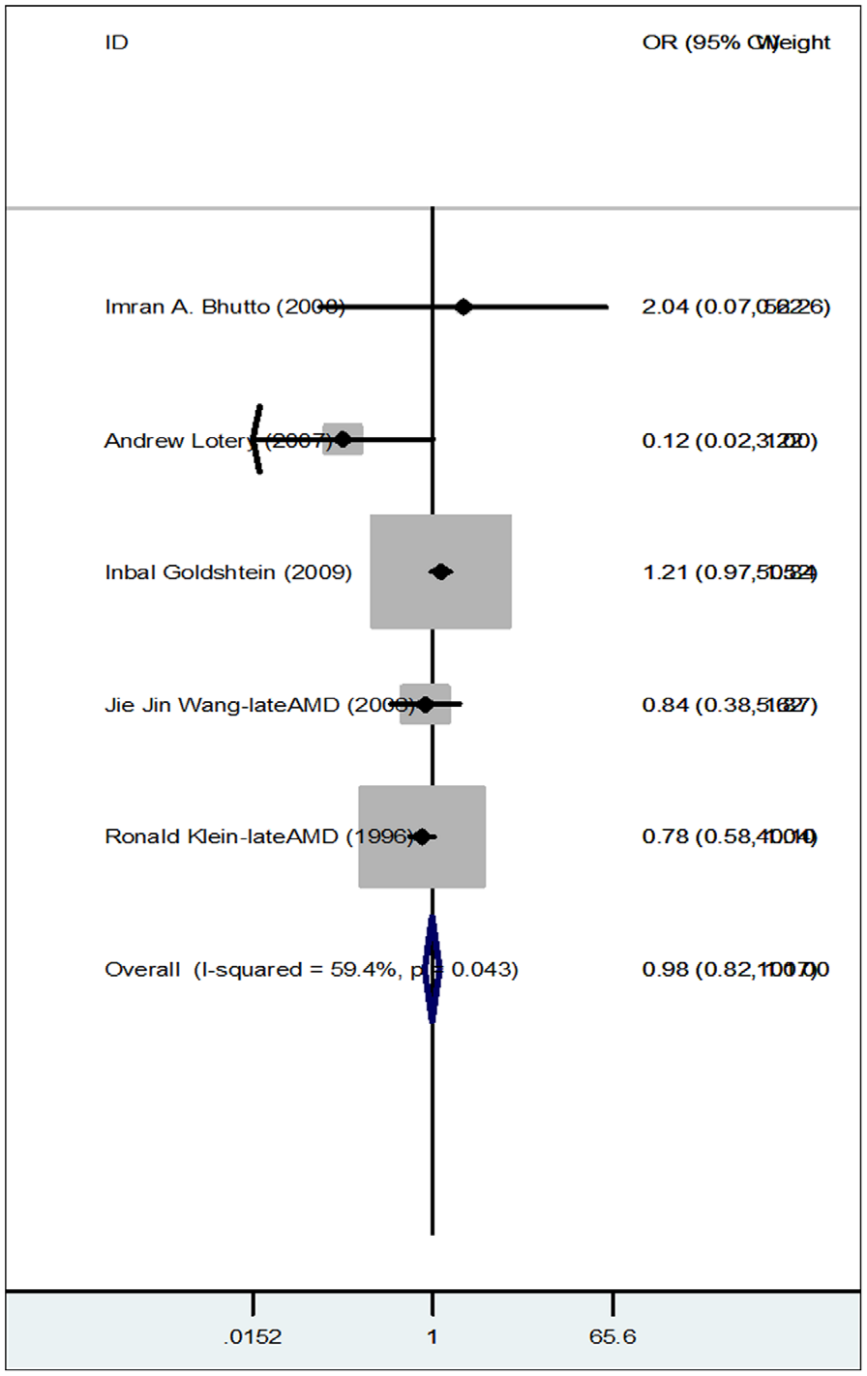
Forest plot showing the results of our meta-analysis. Each study is shown by the point estimate of the OR(the size of the square is proportional to the weight of each study) and 95% CI for the OR(extending lines).

To obtain more information about the results of other epidemiological studies, a meta-analysis was performed. Seven epidemiological studies were identified that provided information regarding the potential association of CNV and asthma. All of the studies analyzed were written in English [18]–[23]. There were two articles that included multiple studies [20], [23], and we treated each study separately. 4054 CNV cases and 109006 healthy controls, for a total of 113060 subjects, were included in the study. When we pooled all seven studies into this meta-analysis, we found no significant association between the risk of AMD and asthma (OR: 0.98; 95% CI:0.82–1.06) (Fig. 1). We also performed Egger’s test to check whether there was publication bias, and it confirmed that in the dominant model, there was an absence of publication bias (Fig. S1).

### An Asthma Rat Model Shows More Fluorescein Leakage from Laser Induced Choroidal Neovascularization Lesions on FA Analysis and More Neovascularization on Histological Analysis than in Rats without Asthma

**Figure 2:**
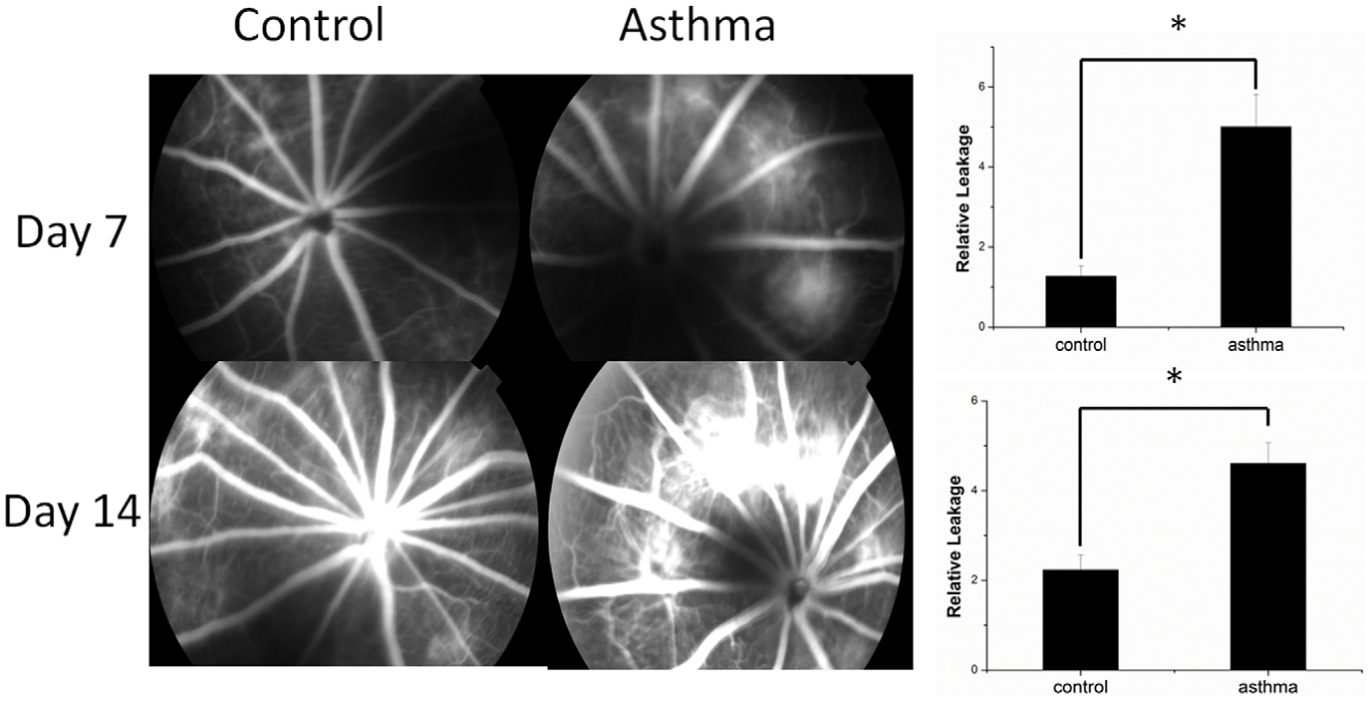
CNV leakage on FA. Angiographic analysis of CNV leakage 7 and 14 days after laser photocoagulation in 5rats with asthma and the control group 5 rats. Rats with asthma developed more CNV leakage on both day 7 (A, B) and day 14 than the rats in the control group (C, D).

The results of our epidemiological study and meta-analysis were inconclusive. Therefore, an animal model was established to provide further analysis. According to the epidemiological study we performed, CNV was related to asthma. Thus, a CNV model was developed in rats with asthma and in rats without asthma as a control. CNV leakage in rats with and without asthma was assessed by fluorescein angiography to see whether rats with asthma were more likely to develop CNV. Using FA performed on day 7 after laser-induced CNV, very few patches of hyper-fluorescence leakage were observed at the lesion site of rats without asthma, but leakage could be seen in rats with asthma, and there was a significant difference (P = 0.001). The leakage distribution at day 14 increased when compared to day 7. There were a total of 33 CNVs in five rats with asthma, while the number of CNV patches in the groups without asthma was 16(P = 0.002) (Fig. 2).

**Figure 3:**
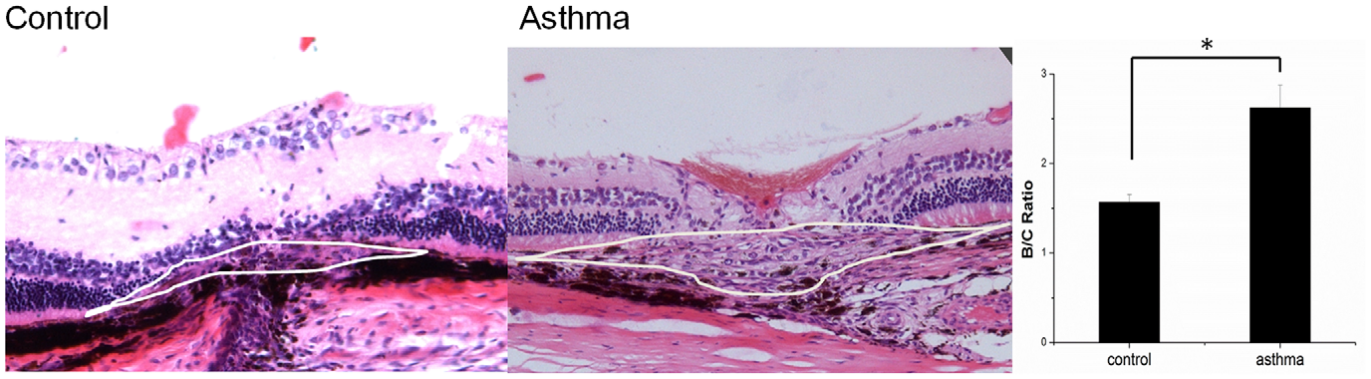
H&E staining of representative areas of CNV. H&E staining of representative areas of CNV at the sites of laser-induced lesions in 5 rats with asthma (A) and the 5 control group (B).Eyes were removed 14 days after laser photocoagulation. Note the presence of laser-induced CNV in each group.

Two weeks after laser photocoagulation, histopathology performed on hematoxylin and eosin stains obtained from paraffin cross-sections confirmed that there was increased invasion in the retina around the laser scars in rats with asthma compared to rats without asthma. There were significant differences in the B/C ratio between the groups with asthma and the control (P = 0.001) (Fig. 3).

### Meta-analysis Demonstrating an Association between C3 and CNV

The relationship between C3 and CNV has been previously reported. However, to obtain a systematic review, a meta-analysis of C3 R102G and CNV was performed. 16 genetic epidemiological studies were previously preformed to analyze the association of CNV and the C3 R102G polymorphism (4 studies were over-lapped) [7], [24]–[26], and all of them were written in English. There were 14 articles published regarding the association between R102G and AMD risk. Because 3 studies were overlapping, we selected the articles with the largest sample size. There were 2 articles that included more than one study [24], [25], and we treated them as separate studies.

**Figure 4:**
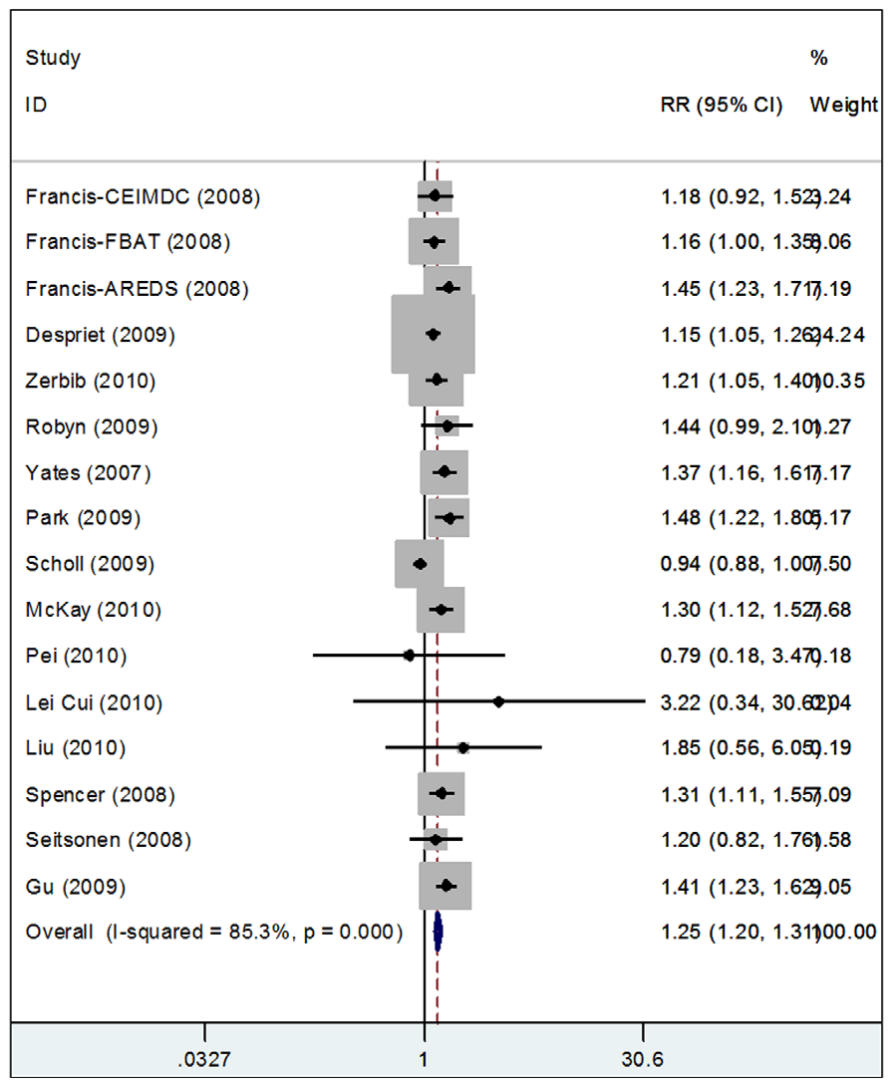
Meta-analysis of C3 R102G and AMD. Forest plot for meta-analysis of association between R102G and AMD risk. Each study is shown by the point estimate of the odds ratio (OR)(the size of the square is proportional to the weight of each study) and 95% confidence interval(CI) for the OR(extending lines).

The genotype effects for GC and GG versus CC genotypes were 1.459 (95% CI: 1.278, 1.667) and 1.471 (95% CI: 1.016, 2.130), respectively. This meant that patients who had GG and GC genotypes were roughly 1.5 times more likely to have AMD than patients with the CC genotype. These results suggested a codominant genetic model in which C3 R102G polymorphism plays an important role in the pathogenesis of AMD (Fig. 4).

### Increased Expression of C3 and VEGF Could Affect the Development of CNV in Rats with Asthma

**Figure 5:**
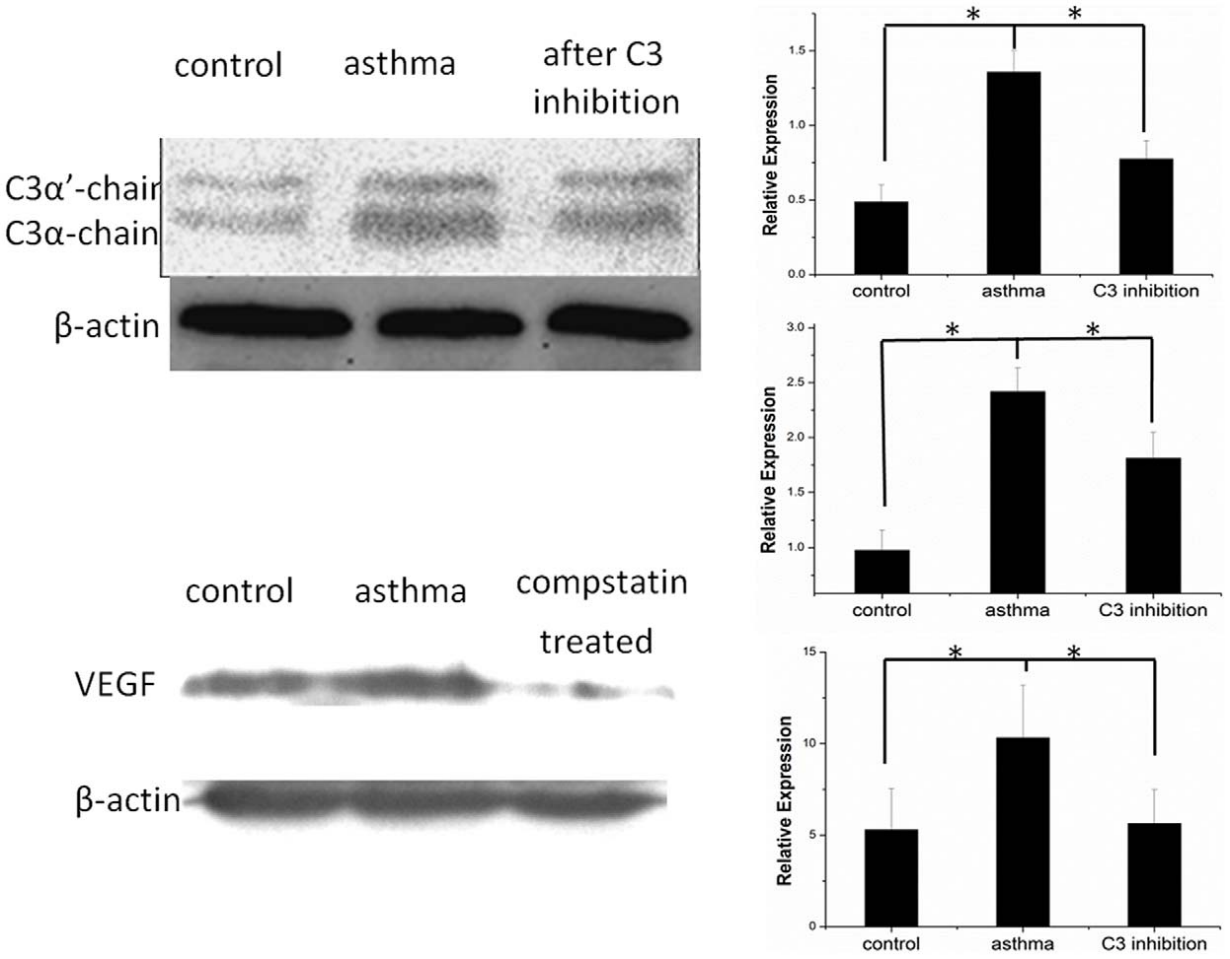
Western blot of C3 and VEGF. Western blot showing C3α’-chain, C3α-chain and VEGF expression in the RPE/choroid layer of rats without asthma, rats with asthma and rats with asthma treated with compstatin 14 days after laser photocoagulation. This experiment was repeated three times.

We next investigated the possible mechanism why rats with asthma developed more CNV than rats without asthma. As mentioned above, C3 and VEGF play fundamental roles in the development of both CNV and asthma. Western blot analysis of C3 and VEGF was performed, with β-actin serving as an internal loading control. We found an increase in the levels of the C3 split products (113 kDa for C3α-chain and 106 kDa for C3α’-chain) and VEGF (51 kDa) in rats with asthma compared to rats without asthma. C3α-chain levels were significantly increased by 2.59 fold (P = 0.006), with C3α’-chain levels increasing 3.00 fold (P = 0.023) and VEGF level increasing 2.87 fold (P = 0.031) (Fig. 5).

### Inhibitory Effect of Compstatin on CNV-associated Down Regulation of C3 and VEGF

Given that the result has shown that CNV growth is associated with C3 and VEGF, either of the following scenarios is plausible: 1) elevated C3 levels induce an increase in VEGF, 2) elevated VEGF levels induce an increase in C3, or 3) C3 and VEGF levels are elevated independently.

**Figure 6:**
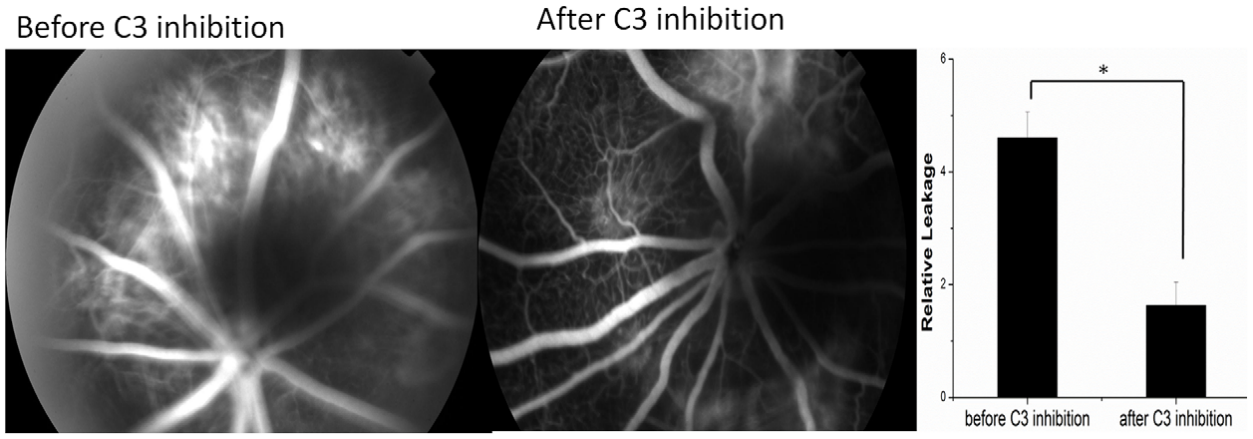
CNV leakage on FA after compstatin injection intravitreally. Angiographic analysis of CNV leakage 7 days after intravitreal compstatin injection. The pathologic leakage in rats with asthma was reduced after treatment.

To gain further insight into the relationship between VEGF and C3 and their effects on CNV in asthma, we investigated the use of compstatin to test the effect of C3 activation in the retina. 7 days after intravitreal injection of compstatin, fluorescein angiography was performed and CNV leakage was analyzed. The number of CNV patches in the rats receiving intravitreal injection was 10 compared with 33 CNV patches in rats that had not received intravitreal injection. Pathologic leakage was significantly reduced after treatment with compstatin (P = 0.03) (Fig. 6). Western blot analysis revealed a significant down regulation in the levels of C3α-chain, C3α’-chain and VEGF by 1.35 (P = 0.001), 1.78 (P = 0.012) and 1.82 (P = 0.044) fold, respectively, in RPE/choroid samples from CNV rats (Fig. 5). Thus, having asthma may provide a source of C3, raising the probability that locally produced C3 may precipitate VEGF release and CNV development [10].

## Discussion

Our epidemiological study demonstrated that CNV was related to asthma. However, the meta-analysis showed a summary OR of 0.98(95%CI: 0.82–1. 06), which was contrary to our results. Further experiments found that in an animal model, rats with asthma had earlier and more extensive neovascularization compared with rats without asthma and C3 and VEGF may have played a fundamental role in this difference.

The association between CNV and asthma was controversial in previous studies. In our epidemiological study, asthma was associated with CNV with a 95% CI of 1.721. Among relevant studies searched, Inbal Goldshtein found a history of asthma was a risk factor for CNV. However, Jie Jin Wang and Ronald Klein were unable to replicate Goldshtein’s findings [20], [23]. After integrating the accumulating evidence, our meta-analysis demonstrated that asthma was not associated with CNV. This result was convinced because no potential publication bias was found. Thus, the results of our epidemiological study and meta-analysis were not consistent. There are several possible explanations for the inconsistency we found. First, there were a limited number of publications available, which included a total of only 7 studies. Therefore, the small sample size used for our meta-analysis could be a reason for the discrepancy. Second, because different studies were carried out in different regions and in different ethnic groups (Imran A. Bhutto in USA, Andrew Lotery in UK, Inbal Goldshtein in Israel, Jie Jin Wang in Australia and Ronald Klein in USA), there could be significant differences in both the environment and inheritance of genes that could potentially bias the results. Different regions and ethnic groups also have variability in the frequency and severity of asthma attacks, which could lead to deviations. Third, according to our epidemiological study, asthma was related to CNV but not to dry AMD. However, in the studies we searched for our meta-analysis, the subgroups CNV and dry AMD were not distinguished. The absence of subgroups, therefore, may be a factor in why different results were obtained.

Our animal model study demonstrated that asthma is a risk factor for the development of CNV, which we speculated could be a consequence of systemic inflammation associated with asthma.

Systemic inflammation induced by asthma and the ocular inflammation that results may explain the relationship between CNV and asthma. An inflammatory response is involved in the development of CNV in both rats and humans [10]. There is strong evidence suggesting that in the pathogenesis of choroidal angiogenesis both systemic and ocular inflammation are involved [20]. In patients with asthma or animal models of asthma, allergens are known to drive allergic airway inflammation and activation of the inflammatory corpuscle resulting in systemic cytokine release.

Activation of the AP has been well documented in the development of CNV. As a point of convergence of the AP, C3 plays an important role in the control of the innate immune system and the inflammatory reaction that has been suggested to contribute in the pathogenesis of CNV. In patients with asthma, increased levels of serum C3 have been described [17]. As a result of systemic C3 elevation, local C3 levels are elevated accordingly resulting in local activation of the complement pathway.

Our study showed higher levels of C3 and VEGF in rats with asthma compared to rats without asthma. To determine whether the accumulation of C3 induced VEGF or if the opposite is true, a C3 inhibitor–compstatin was used. We found that C3 and VEGF protein levels decreased at the same time. According to Puran S. Bora, C3 activity is critical for the formation and deposition of the terminal membrane attack complex (MAC) on RPE and choroid cells, which leads to transient changes in the permeability of cell membrane. Changes in the permeability of the cell membrane lead to induction and release of VEGF, which eventually leads to the development of CNV [3] (Fig. 7).

Compstatin is a potent inhibitor of the complement system which inhibits cleavage of complement protein C3 [27]. Although it was tested to be inactive against C3 from lower mammals in a Surface Plasmon Resonance (SPR) chips measurement experiment [28], we believe in its functions intravitreally, still. As SPR only analyzes the binding process, its affinity for compstatin could be affected in vivo by other small molecules. And because of its specificity of the detection is not high enough, further studies such as IP are needed to test their result. In vivo studies are needed, too.

According to our study, asthma is a risk factor in the development of CNV because people with asthma are more likely to have CNV. This information could potentially lead to earlier screening for CNV in patients with asthma.

In conclusion, CNV is associated with asthma in our epidemiological study. However, previous studies show conflicting conclusions. We also found that asthma may affect the development of CNV, by increasing the protein levels of C3 and VEGF in the RPE/choroid layers, as observed in a rat model of allergic asthma. This study was the first to systematically investigate the relationship between CNV and asthma and may provide a better understanding of the disease. This knowledge may help to advance the potential for screening among asthma patients in clinical practice.

## Materials and Methods

### Epidemiological Study

17 hospitals located in 9 different provinces in China enrolled 545 patients and 509 healthy controls from May 2010 to March 2011. Patients over the age of 50 and diagnosed with AMD according to Fluorescein angiography (FFA) and/or Optical Coherence Tomography (OCT) were included as long as they had no other retinal diseases. Patients with pathological myopia, macular dystrophy, central serous chorioretinopathy, retinal vein occlusion, diabetic retinopathy or uveitis were excluded. All healthy patients were examined by FA and/or OCT to exclude CNV. A history of asthma was surveyed and the diagnoses were made by respiration experts according the Global Initiative for Asthma Guidelines 2007.

### Meta-analysis

#### Publication search

Meta-analysis was performed as previously described [29]. Relevant studies were found by searching the following keywords: “asthma” and “AMD” or “CNV” in Pub Med, Medline and Web of Science databases (updated to Oct 18, 2011). All of the selected studies were retrieved, with their references checked for other relevant publications as well. No minimum number of patients was required for meta-analysis. Different sub-studies in the same article were treated as separate studies. In this meta-analysis, we selected only those studies published in English and only when the full text articles were available. For each paper, we extracted the following information into a table: first author, year of publication, number of cases and controls, and crude odds ratio (OR). Two authors (Yaoyao Sun and Peng Zhou) read the identified articles carefully and assessed them independently. For any discrepancies in their eligibility, they were adjudicated by Xiaoxin Li.

#### Statistical methods

The OR with 95% confidence intervals (CI) was calculated to evaluate the association strength between a history of asthma and CNV risk. Both fixed-effects (the Mantel-Hzenszel method) and random-effects (Der Simonian and Laird method) models were chosen. Cochran’s Q statistic was used for determining the statistical significance of heterogeneity. If the p-value was less than 0.10, we selected the random-effects model otherwise the fixed-effects model was used. We used forest plots to describe the results from separate studies as well as for a summary containing all of the results. We used Egger’s tests to test the potential for publication bias, and if P<0.05, the publication bias was considered to be statistically significant. Forest plots were produced to graphically present significant findings. Funnel plots were used to explore the potential for publication bias (PRISMA checklist) [30].

Because few studies investigating the association between C3 SNPs and asthma were found, we analyzed only the association between C3 SNPs R102G polymorphisms and AMD because this SNP has been the most commonly studied one. In order to obtain additional results, the following four different types of OR were calculated: (1) CG versus CC genotype, (2) GG versus CC genotype, (3) CG plus GG versus CC genotype (which was the dominant model) and (4) GG versus CG plus CC genotype (which was the recessive model).

All of the statistical calculations were conducted using Stata/Se version 11.0 software (Stata Corporation, College Station, TX).

### Animals

Specific, pathogen-free female Brown Norway (BN) rats weighing 150±10 g were purchased from the Laboratory Animal Center, Peking University People’s Hospital. Animal care and experiments were conducted under institutional guidelines and food and tap water were given ad libitum.

#### Establishment of an asthmatic rat model

On days 0 and 5, 10 rats in the asthma group were actively sensitized by intraperitoneal (i.p.) injection of 1mg ovalbumin (OVA, Sigma-Aldrich, St. Louis, MO, USA) emulsified with 40 mg Imject Alum (Pierce, Rockford, IL USA) in a total volume of 1ml. In order to elicit an airway allergic response, these rats were placed in a chamber made of plastic and challenged with an OVA aerosol (1% (w/v) in 0.9% NaCl) nebulized by an ultrasonic nebulizer (PARI BOY, PARI GmbH, Sternberg, Germany) twice a day for 1hour from days 6 to 14.10 rats in the negative control group were sham immunized with 0.9% NaCl and then challenged with an aerosol of 0.9% NaCl at the same frequency as the asthma group [31], [32]. HE staining of lung in asthma was carried out (Fig. S2), and the status asthmaticus in asthma rats was recorded by video (Video S1, Video S2).

#### Induction of CNV

CNV was induced by laser photocoagulation (532 nm, 150 mW, 100 ms, 100 µm).

(Coherent 130SL, Coherent, Santa Clara, CA, USA) performed on day 15 when the asthma model was thought to have already been established. 8 lesions were made on one eye of each rat, and the other eye was used as the control.

#### Intravitreally injection of compstatin

To test the effect of suppressing complement activation in the retina of rats with asthma, 20 µg compstatin, a small cyclic synthetic peptide (Tocris Bioscience, Ellisville, Missouri, USA), was intravitreally injected into 5 rats 7 days after CNV was induced. Compstatin was dissolved in 1ml of 0.9% NaCl solution per 100 µg, filtered and intravitreally injected with a micro injector while the rats were anesthetized with 0.5 ml 10% chloral hydrate injected i.p.

### The Measurement of CNV

#### Fluorescein angiography

Fluorescein angiography (FA) was performed on day of 7 and 14 after laser photocoagulation using a digital imaging system (TOPCON 50DX, Topcon, Tokyo, Japan) as described previously [33]. 0.2 ml of 5% fluorescein was given by i.p. after the rats were anaesthetized using the method described above and FA was done with pupil dilation. Both early-phase (1 minute after injection) and late-phase (5 minutes after injection) fundus angiograms were analyzed [34]. Fluorescein angiograms were evaluated quantitatively and the leakage area for each lesion was measured using ImageJ software, a custom programmed macro provided by the National Institutes of Health (NIH).

#### Histological analysis and CNV size measurement

14 days after laser photocoagulation, the eyeballs of the experimental eye were removed and were fixed in eyeball fixing solution for 24 hours at room temperature. After removal of the anterior segments, the posterior eyecups were embedded in paraffin. Sagittal sections of 6µm were cut through the center of the eye at the site of laser photocoagulation. The sections were stained by hematoxylin and eosin and assessed by light microscopy (LEICA DFC 300FX, Leica, Solms, Germany). A computer-assisted image analysis system was used to estimate neovascularization based on the B/C ratio (B stands for the thickness between the bottom of the pigmented choroidal layer and the top of the neovascular membrane, while C stands for the thickness of the intact-pigmented choroid next to the lesion [35]. Measurements were performed on four sections from each laser photocoagulation site.

### Western Blot

Fourteen days after laser photocoagulation, we removed the experimental eyeballs and extracted protein. Total protein was extracted from the pooled RPE/choroid layers and protein concentration was measured using the Bio-Rad assay kit (Bio-Rad, Hercules, CA, USA). Equal amounts of protein (30–60 µg) were resolved on 12% (for C3 antibody) or 6% (for VEGF and β-actin antibodies) Tris-HCl polyacrylamide gels and then transferred to a PVDF blotting membrane (Millipore, Billerica, MA, USA). After blocking, membranes were incubated with specific antibodies for C3 (Santa Cruz, CA, USA), VEGF (Abcam, Cambridge, MA, USA) and β-actin (Abcam, Cambridge, MA, USA). After incubation with peroxidase-conjugated goat anti-rabbit or anti-mouse secondary antibodies (ZSGB-Bio, Beijing, China), protein bands were visualized by chemiluminescence (Pierce, Rockford, IL, USA). This experiment was repeated three times, and similar results were obtained each time [36], [37].

### Statistical Analysis

All of the experiments were repeated three times and the data were presented as mean±SEM. Data were analyzed using Student’s T test. p<0.05 was considered statistically significant. All data analyses were performed with SPSS 17.0 (Chicago, IL, USA).

## Supporting Information

Figure S1 **Publication bias of the meta-analysis.** The Begg’s funnel plot of studies included in the meta-analysis between asthma and AMD(A) and between C3 R102G and AMD(dominant model,B). The vertical axis represents log[OR] and the horizontal axis means the standard error of log[OR]. Horizontal line and sloping lines in funnel plot represent random effect summary OR and expected 95%CI for a given standard error, respectively. Area of each circle represents the contribution of each study to the pooled OR.
(TIF)

Figure S2 **HE stainings of lung in asthma group and control group.** Remarkble infiltration of inflammatory cells (including eosinophils and neutrophils) around the bronchioles with the destruction of epithelium, accumulation of inflammatory debris could be seen in asthma rats(A) but not in control rats(B).
(TIF)

Video S1 **showed the status asthmaticus in asthma rats.**
(WMV)

Video S2 **showed the normal performance in the control group.**
(WMV)
