# Supplementary figures and images for: Is Asthma Related to Choroidal Neovascularization?
Source: PLoS One. 2012 May 2;7(5):e35415. doi: 10.1371/journal.pone.0035415 (PMC3342271; doi:10.1371/journal.pone.0035415)

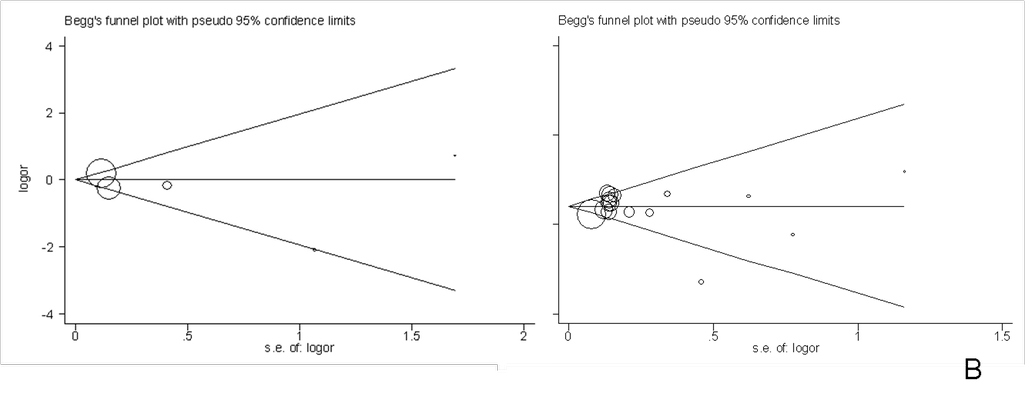

Supplement: Figure S1 — Publication bias of the meta-analysis. The Begg’s funnel plot of studies included in the meta-analysis between asthma and AMD(A) and between C3 R102G and AMD(dominant model,B). The vertical axis represents log[OR] and the horizontal axis means the standard error of log[OR]. Horizontal line and sloping lines in funnel plot represent random effect summary OR and expected 95%CI for a given standard error, respectively. Area of each circle represents the contribution of each study to the pooled OR. (TIF) [file pone.0035415.s001.tif]

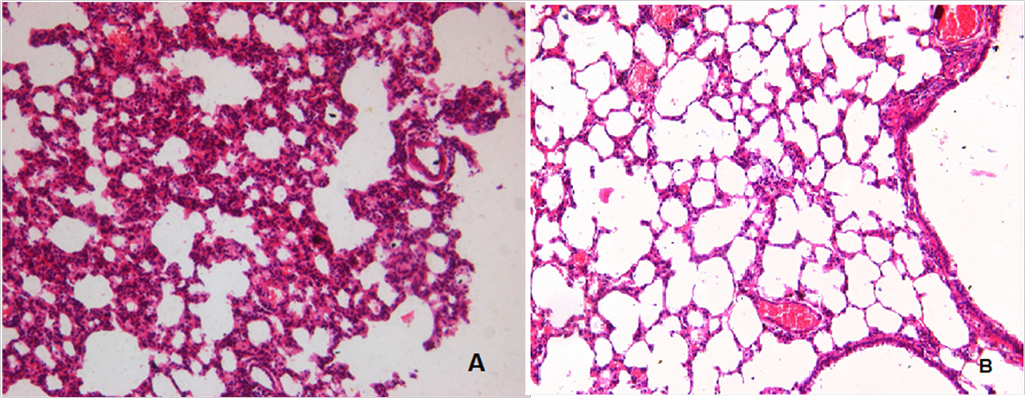

Supplement: Figure S2 — HE stainings of lung in asthma group and control group. Remarkble infiltration of inflammatory cells (including eosinophils and neutrophils) around the bronchioles with the destruction of epithelium, accumulation of inflammatory debris could be seen in asthma rats(A) but not in control rats(B). (TIF) [file pone.0035415.s002.tif]
